# The association of race with time to severe liver disease diagnoses

**DOI:** 10.1371/journal.pone.0334016

**Published:** 2025-10-14

**Authors:** Andrew D. Schreiner, Jingwen Zhang, Mulugeta Gebregziabher, Justin Marsden, Patrick D. Mauldin, Don C. Rockey

**Affiliations:** 1 Department of Medicine, Medical University of South Carolina, Charleston, South Carolina, United States of America; 2 Department of Public Health Sciences, Medical University of South Carolina, Charleston, South Carolina, United States of America; Medizinische Fakultat der RWTH Aachen, GERMANY

## Abstract

**Background:**

Evidence suggests that there are racial differences in liver fibrosis progression for patients with chronic liver disease (CLD). We examined the association of Black race with the time to diagnosis of severe liver disease outcomes in primary care patients.

**Methods:**

We captured electronic health record data from a primary care clinic between 2012−2021. Race, categorized as Black and non-Black, was the primary exposure. The outcome was the occurrence of a severe liver event identified by ICD-9/10 codes, defined as a composite of cirrhosis, complications of cirrhosis, hepatocellular carcinoma, and liver transplantation. Cox regression models evaluated the association of Black race with the time to severe liver outcomes while adjusting for potentially confounding covariates.

**Results:**

The cohort included 20,828 patients of whom 43% identified as Black and 14% had a known diagnosis of CLD during follow-up. Of all patients, 3% received a diagnosis code for a severe liver event. In an unadjusted Cox regression model, Black race was associated with an increased hazard of a severe liver event (HR 1.32; 95%CI 0.98–1.34), but after adjusting for known CLD, baseline fibrosis risk, demographic, and comorbidity variables, Black race was associated with a significantly lower hazard of a severe liver outcome (HR 0.68; 95%CI 0.57–0.81).

**Conclusions:**

After adjusting for potentially confounding covariates, Black race was associated with a longer time to a severe liver disease diagnosis. This finding raises the possibilities of delayed cirrhosis detection or differences in liver fibrosis progression by racial identifiers.

## Introduction

Chronic liver disease (CLD), with etiologies including viral hepatitis (B and C), alcohol-related liver disease (ALD), and metabolic dysfunction-associated steatotic liver disease (MASLD, formerly nonalcoholic fatty liver disease [NAFLD]), affects an estimated 1.5 billion people globally [1]. Complications from CLD, comprising cirrhosis, portal hypertension and its associated clinical features, hepatocellular carcinoma, and liver failure requiring liver transplantation, have increased substantially over the past 20 years [1]. During this time, CLD has emerged as the fifth leading cause of death in Americans 35–64 years of age, and the 9^th^ leading cause of death in the U.S [1–3]. Despite substantial progress in the availability and efficacy of therapies for viral hepatitis C (HCV) and B (HBV), the burden of CLD continues to grow, in part due to the increasing prevalence of alcohol use and the ongoing epidemic of obesity, diabetes, and metabolic syndrome accompanying MASLD [1,4].

Though many patients are affected by CLD, only some progress to develop advanced fibrosis, cirrhosis, and other severe liver disease outcomes [5,6]. Previous studies have identified risk factors for severe liver disease in patients with HCV and MASLD, including advanced age, male sex, smoking, and concomitant alcohol use [6,7]. Studies of patients with HCV have demonstrated clear differences in fibrosis progression and response to therapy, where patients identified as Black had lower odds of developing advanced fibrosis or cirrhosis [8–10]. In MASLD, patients identified as Black were less likely to have advanced fibrosis than their white counterparts [11,12]. However, these studies have been limited by small sample sizes, focus on singular CLD entities, and some had few subjects that identified as Black.

In this study, we aimed to evaluate the association of identifying as Black with the time to occurrence of a severe liver disease diagnosis by adjusting for potentially confounding covariates in a large, diverse, retrospective cohort using data form a robust electronic health record (EHR). We hypothesized that patients identified as Black would have a reduced risk of liver fibrosis progression and therefore would have a longer time to severe liver disease outcomes compared to non-Black patients. We recognize that investigation of race and disease is challenging due to the multi-faceted construct that race represents, and racial identifiers often incorporate environmental factors, socioeconomic status, discrimination, and in many cases, restricted access to care [13]. Despite these challenges, the association of race and ethnicity with health outcomes may provide important signals for exploring genetic predictors of disease, and provide a better understanding of its pathogenesis.

## Materials and methods

### Study design, setting, and data source

This retrospective cohort study of EHR data comes from a primary care practice at the Medical University of South Carolina (MUSC) from 2012 to 2021. Data were collected from the EPIC^©^ EHR and the Enterprise Data Warehouse at MUSC. Data was coded with separation of identifiers from data points and accessed most recently January 15, 2025. This study was approved by the MUSC Institutional Review Board (IRB, Pro00056541). The MUSC IRB waived the requirement for informed consent.

### Patients

Patients with two sets of liver chemistry and complete blood count laboratory results were considered for inclusion [14]. This criterion ensured that only patients with at least two data points were evaluated. This dataset was originally constructed for another study, which required the aspartate (AST) and alanine (ALT) aminotransferase values to be both < 350 IU/L and the platelet count to have been recorded within six months of the AST and ALT results [15]. Patients with a previous diagnosis of cirrhosis, hepatocellular carcinoma, or who had a liver transplant prior to the first set of lab results were excluded (S1 Table). Follow-up during the study period began after the first laboratory values and continued until the occurrence of a primary outcome or the end of the study period (December 31, 2021).

### Outcome

The primary outcome was the time to a severe liver disease outcome, defined as having a diagnosis of cirrhosis, a complication of cirrhosis (including ascites, esophageal varices, hepatic encephalopathy, hepatopulmonary syndrome, hepatorenal syndrome, or clinical portal hypertension), hepatocellular carcinoma, or liver transplantation [16–18]. Outcomes were deemed to have occurred when a severe liver disease ICD-9/10 code was recorded during the study period (S1 Table). Combinations of ICD-9/10 codes were selected based on previous studies that evaluated sensitivity and specificity of different combinations of codes for detecting severe liver outcomes from administrative data [16–18]. The collection of codes used, specifically those incorporating the complications of cirrhosis, ensured high sensitivity for cirrhosis detection in the EHR.

### Primary predictor variable

Self-identified race, a categorical variable structured as Black/ non-Black, was the primary predictor variable of interest. A low distribution of clinic patients were identified as non-Black or non-White (<3%), and since our question specifically regarded severe liver disease risk in patients identified as Black, we chose to dichotomize the race variable.

### Covariates

Diagnoses of CLD at baseline and during follow-up were included as covariates. We structured CLD as a dichotomous categorical variable (Yes/ No) and as a more specific, 5-level categorical variable (Alcohol/ MASLD/ Viral hepatitis/ Other/ None) for a sensitivity analysis. The “Other” category included autoimmune hepatitis, primary biliary cholangitis, hemochromatosis, Wilson’s disease, and α-1 anti-trypsin deficiency. Patients with more than 1 CLD diagnosis were attributed to a viral, alcohol, MASLD, or other chronic liver disease in that order of priority. CLD variable status was assigned using ICD-9/10 codes identified any time before or during follow-up and was categorized as “None” in patients receiving a severe liver disease diagnosis without a CLD documented by the time of the severe liver outcome (S1 Table).

Advanced fibrosis risk at the beginning of follow-up was included as a covariate. Fibrosis-4 Index (FIB-4) scores were calculated (FIB-4=[Age x AST]/[Platelets x √ALT]) for each patient in the cohort at the beginning of follow-up with advanced fibrosis risk categorized as low- (FIB-4 < 1.3), indeterminate- (1.3 ≤ FIB-4 < 2.67), and high-risk (FIB-4 ≥ 2.67) [19–21]. This covariate was included to adjust for advanced fibrosis risk for each patient at the beginning of follow-up.

We also examined potentially confounding variables comprising demographic and comorbidity factors. Age was included as a continuous variable while categorical variables included sex (Female/Male), marital status (Married/Unmarried), and smoking history (Yes/No). Accurate data regarding social determinants of health were limited, so the zip code of the patient’s residence was matched to 2010 Census data as a proxy for poverty status. The poverty variable was dichotomous and categorized by whether the zip code of residence had ≥ 25% of its residents below the federal poverty level. We also included a variable of remote residence, a dichotomous variable categorized by whether a patient lived ≥ 50 miles from the primary care clinic. Body mass index (BMI) was included as a continuous variable. Comorbidities including hypertension, diabetes mellitus, hyperlipidemia, cardiovascular disease (CVD), hypothyroidism, chronic kidney disease (CKD), and alcohol use disorder (AUD) were categorical variables (Yes/No) identified during follow-up by ICD-9/10 codes using Elixhauser coding algorithms [22,23].

### Statistical analysis

Univariate analyses were performed to describe the distribution of covariates in the cohort. Cohort characteristics were compared by Black/ non-Black status and by severe liver disease outcome (Yes/No), with two sample t-tests for continuous variables and Chi square tests for categorical variables. We created Kaplan-Meier (KM) curves to display the severe liver disease free survival of patients by racial category and to test the assumption of proportional hazards. A series of Cox regression models were developed with race as the primary predictor variable and the time to a severe liver disease as the outcome (Models 1−5). Since the KM curves from the raw data crossed, we accounted for the non-proportionality of the hazards by fitting a time-stratified Cox model (where the strata variable was created as binary with time before and time after the crossing of the KM curves) [24]. By stratifying the analysis by time, it divided the sample into sub-groups, re-estimated the models, and allowed for differences in the baseline hazard function between the subgroups. Model 1 described the unadjusted association between race and time to severe liver outcomes. Model 2 incorporated CLD as a covariate to adjust for previously known CLD. Model 3 added to Model 2 by also adjusting for FIB-4 fibrosis risk at baseline. Model 4 built upon Model 3 by also adjusting for sex, marital status, smoking history, impoverished zip code status, and remote residence. Finally, a model (Model 5) adjusting for the other potentially confounding covariates was developed and included BMI, and comorbidities (diagnoses of hypertension, diabetes, hyperlipidemia, CVD, hypothyroidism, CKD, and AUD). All variables were specified *a priori*. Models were assessed for goodness of fit. We tested for interactions and assessed for multicollinearity by variance inflation factor. Observational studies that attempt to evaluate causality between an exposure and an outcome are subject to unmeasured confounding. To address this problem, we also calculated an E value to determine the minimum strength of association that an unmeasured confounder would need to have on race and time to severe liver disease to explain away the exposure-outcome association. The E value is calculated by a formula using the risk ratio (RR), E-value = RR + √(RR x [RR-1]) [25]. We performed sensitivity analyses which included developing adjusted Cox models with each covariate added one by one. We also performed sub-analyses where we divided the dataset into patients with a known CLD diagnosis and those without a CLD diagnosis during follow-up. We developed unadjusted and fully adjusted Cox regression models for these subsets. SAS 9.4 (Cary, NC) was used for all statistical analyses.

## Results

**Fig 1:**
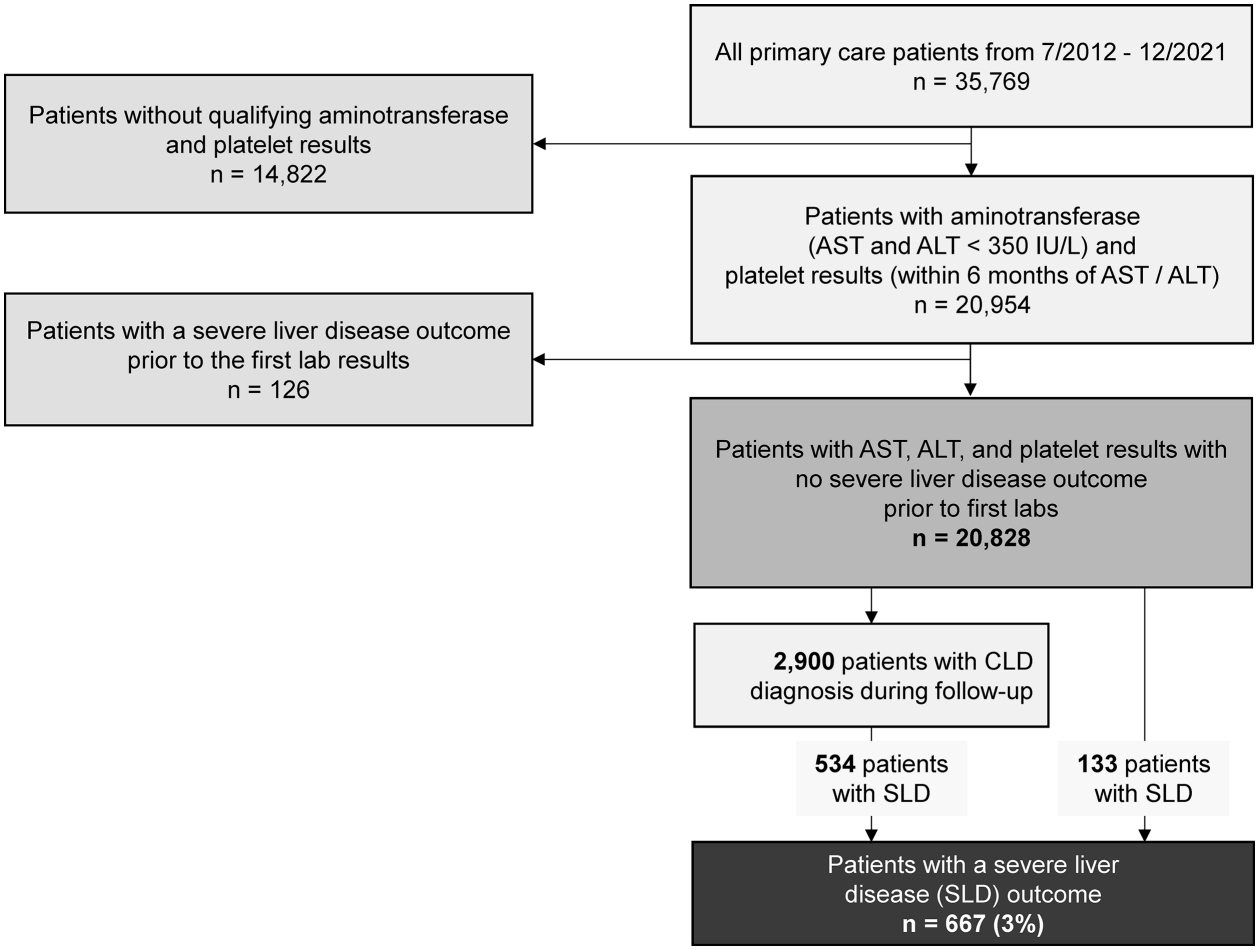
Consort diagram for the cohort. ALT = alanine aminotransferase. AST = aspartate aminotransferase. CLD = chronic liver disease.

The cohort included 20,828 patients (**Fig 1**) with a mean age of 52 years and of whom 43% identified as Black and 65% were female (**Table 1**). During the study, 2,900 (14%) patients had a CLD diagnosis with alcohol as the most common CLD etiology (5%), closely followed by viral hepatitis (4%), and MASLD (3%). Compared to their non-Black counterparts, patients identified as Black had higher proportions of CLD (15% vs. 13%, p < 0.01), ALD (5.4% vs. 4.2%, p < 0.01), viral hepatitis (3.2% vs. 1.8%, p < 0.01), and other CLD (2.4% vs. 0.7%, p < 0.01). Of the cohort, 7% of patients had FIB-4 scores at high-risk for advanced fibrosis at baseline, and the proportion of high-risk FIB-4 scores in Black patients was 8% and 7% in non-Black patients. Patients identified as Black were slightly younger, and a higher proportion were female, smoked, resided in an impoverished zip code, and were diagnosed with hypertension, diabetes, CVD, CKD, and AUD (**Table 1**).

**Table 1:** Characteristics of the primary care study sample overall and by race.

	Overall	Race		
		Black	Non-Black	p-value
Characteristics	n = 20,828	n = 9,022	n = 11,806	
**Chronic Liver Disease Diagnosis (%)**				
CLD Overall	2,900 (14%)	1,377 (15%)	1,523 (13%)	< 0.01^*^
Alcohol	1,136 (5%)	540 (6%)	596 (5%)	< 0.01^*^
MASLD	717 (3%)	207 (2%)	510 (4%)	< 0.01^*^
Viral	753 (4%)	413 (5%)	340 (3%)	< 0.01^*^
Other	294 (1%)	217 (2%)	77 (1%)	< 0.01^*^
**Fibrosis-4 Index Scores at Baseline**				< 0.01^*^
FIB-4 < 1.3	13,146 (63%)	5,763 (64%)	7,383 (63%)	
1.3 ≤ FIB-4 < 2.67	6,091 (29%)	2,528 (28%)	3,563 (30%)	
FIB-4 ≥ 2.67	1,591 (7%)	731 (8%)	860 (7%)	
**Demographics, n (%)**				
Age, mean (SD)	52 (17)	51 (17)	53 (17)	< 0.01^†^
Sex				< 0.01^*^
Female	13,470 (65%)	6,139 (68%)	7,331 (62%)	
Male	7,358 (35%)	2,883 (32%)	4,475 (38%)	
Unmarried	11,513 (55%)	6,671 (74%)	4,842 (41%)	< 0.01^*^
Poverty status	6,556 (31%)	4,000 (44%)	2,556 (22%)	< 0.01^*^
Remote residence	2,704 (13%)	1,035 (12%)	1,669 (14%)	< 0.01^*^
Smoking history	2,429 (12%)	1,391 (15%)	1,038 (9%)	< 0.01^*^
**Comorbidities (%)**				
BMI, mean (SD)	29.7 (7.9)	31.5 (8.9)	28.3 (6.8)	< 0.01^†^
Hypertension	13,977 (67%)	7,186 (80%)	6,791 (58%)	< 0.01^*^
Diabetes	6,141 (30%)	3,940 (44%)	2,201 (19%)	< 0.01^*^
Hyperlipidemia	11,774 (57%)	5,152 (57%)	6,622 (56%)	0.14^*^
CVD	6,456 (31%)	3,252 (36%)	3,204 (27%)	< 0.01^*^
Hypothyroidism	3,683 (18%)	1,076 (12%)	2,607 (22%)	< 0.01^*^
CKD	3,879 (19%)	2,486 (28%)	1,393 (12%)	< 0.01^*^
Alcohol use disorder	1,896 (9%)	1,004 (11%)	892 (8%)	< 0.01^*^

*Chi square test. † Two sample t-test. BMI = body mass index. CKD = chronic kidney disease. CLD = chronic liver disease. CVD = cardiovascular disease. MASLD = metabolic dysfunction-associated steatotic liver disease. SD = standard deviation.

Patients were followed for a mean 6.6 (± 2.6) years, and 667 (3%) patients had a severe liver disease outcome during the study period (**Table 2**). Of the 2,900 cohort patients with a previously known CLD diagnosis, 534 (18%) had a severe liver outcome; 133 patients experienced a severe liver disease outcome before receiving a formal CLD diagnosis. A higher proportion of patients experiencing a severe liver disease outcome had indeterminate- (32% vs. 29%, p = 0.01) and high-risk FIB-4 scores (47% vs. 6%, p < 0.01) at baseline compared to patients without a severe liver disease outcome. Higher proportions of patients experiencing a severe liver disease outcome were older, male, unmarried, smoked, lived in an impoverished zip code, and had diagnoses of hypertension, diabetes, CVD, CKD, and AUD compared to patients without a severe liver outcome (**Table 2**).

**Table 2:** Univariate analysis of cohort characteristics by severe liver disease diagnosis.

	Overall	Severe Liver Disease	No Severe Liver Disease	
Characteristics	n = 20,828	n = 667	n = 20,161	p-value
Race				0.08^*^
Black	9,022 (43%)	311 (47%)	8,711 (43%)	
Non-Black	11,806 (57%)	356 (53%)	11,450 (57%)	
Chronic liver disease	2,900 (14%)	534 (80%)	2,366 (12%)	< 0.01^*^
Alcohol	1,136 (5%)	182 (27%)	954 (5%)	< 0.01^*^
MASLD	717 (3%)	83 (12%)	634 (3%)	< 0.01^*^
Viral	753 (4%)	236 (35%)	517 (3%)	< 0.01^*^
Other	294 (1%)	33 (5%)	261 (1%)	< 0.01^*^
Fibrosis-4 Index Score at Baseline				
FIB-4 < 1.3	13,146 (63%)	135 (20%)	13,011 (64%)	< 0.01^*^
1.3 ≤ FIB-4 < 2.67	6,091 (29%)	216 (32%)	5,875 (29%)	0.01^*^
FIB-4 ≥ 2.67	1,591 (8%)	316 (47%)	1,275 (6%)	< 0.01^*^
Age, mean (SD)	52.1 (17.4)	55.4 (12.4)	52.0 (17.5)	< 0.01^†^
Sex				< 0.01^*^
Female	13,470 (65%)	319 (48%)	13,151 (65%)	
Male	7,358 (35%)	348 (52%)	7,010 (35%)	
Married	9,315 (45%)	212 (32%)	9,103 (45%)	< 0.01^*^
Poverty status	6,556 (32%)	254 (38%)	6,302 (31%)	< 0.01^*^
Remote residence	2,704 (13%)	111 (17%)	2,593 (13%)	0.04
Smoking history	2,429 (12%)	177 (27%)	2,252 (11%)	< 0.01^*^
BMI, mean (SD)	29.7 (7.9)	28.2 (7.0)	29.7 (7.9)	< 0.01^†^
Hypertension	13,977 (67%)	555 (83%)	13,422 (67%)	< 0.01^*^
Diabetes	6,141 (30%)	322 (48%)	5,819 (29%)	< 0.01^*^
Hyperlipidemia	11,774 (57%)	355 (53%)	11,419 (57%)	0.08^*^
CVD	6,456 (31%)	287 (43%)	6,169 (31%)	< 0.01^*^
Hypothyroidism	3,683 (18%)	106 (16%)	3,577 (18%)	0.22^*^
CKD	3,879 (19%)	277 (42%)	3,602 (18%)	< 0.01^*^
Alcohol use disorder	1,896 (9.1%)	331 (49.6%)	1,565 (7.8)	< 0.01^*^

*Chi square test. † Two sample t-test. BMI = body mass index. CKD = chronic kidney disease. CVD = cardiovascular disease. MASLD = metabolic dysfunction-associated steatotic liver disease. SD = standard deviation.

**Fig 2:**
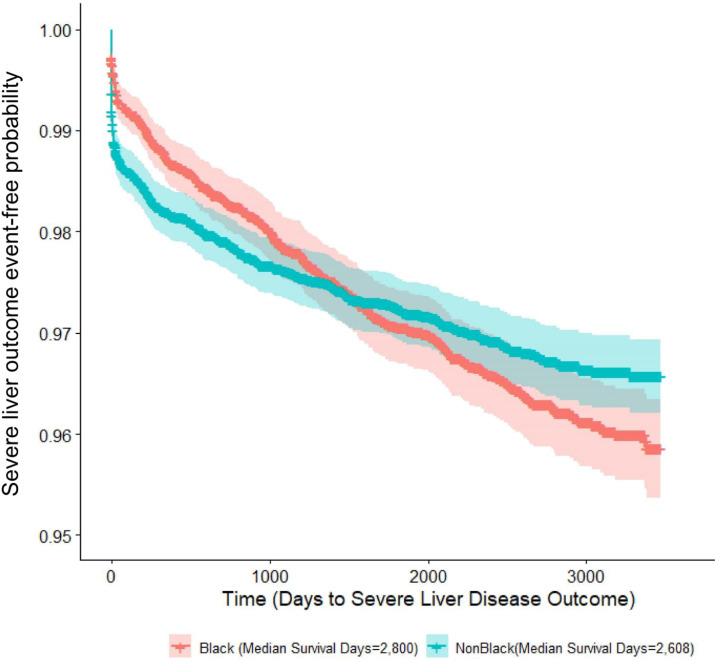
Kaplan Meier survival curve for the outcome of time to a severe liver disease diagnosis (cirrhosis, complication of cirrhosis, or hepatocellular carcinoma) by race.

The KM curves demonstrated that over the first 3 years of follow-up, Black patients were less likely that non-Black patients to be diagnosed with a severe liver disease outcome (**Fig 2**). However, subsequently, Black patients were more likely to develop a severe liver disease outcome. This analysis, which showed that the curves intersect, violated the assumption of proportional hazards [26,27].

We next performed a series of Cox regression analyses, stratified by time, adjusting for confounding variables that might influence the association between race and severe liver outcomes. In the unadjusted model, identifying as Black was associated with an increased risk of severe liver disease (HR 1.32; 95%CI 1.13–1.54, Model 1, **Table 3**). After adjusting for known diagnoses of CLD, there was no significant relationship between Black race and severe liver outcomes (HR 1.11; 95%CI 0.95–1.30; Model 2). The next Cox model also adjusted for baseline FIB-4 risk, which demonstrated no significant association between identifying as Black and time to a severe liver disease outcome (HR 0.93; 95%CI 0.80–1.09, Model 3). The next model (Model 4) also adjusted for demographic variables and identifying as Black was not associated with time to a severe liver disease outcome (HR 0.90; 95%CI 0.76–1.06). After adjusting for known CLD, baseline FIB-4 risk, and potentially confounding demographic and comorbidity covariates, identification as Black race was associated with significantly a lower risk of developing a severe liver outcome (HR 0.68; 95%CI 0.57–0.81) compared to non-Black patients (Model 5). The E-value for this association between identifying as Black and the time to a severe liver disease diagnosis was 2.30 (95%CI 1.76–2.90). In evaluating the sequential Cox regression models with all of the covariates chosen *a priori* added to models one by one, the addition of the hypertension variable is the first model to demonstrate a significant association between identifying as Black and a lower risk of a severe liver disease outcome (HR 0.84; 95%CI 0.71–1.00; S2 Table). In the subset of patients with a known CLD diagnosis during follow-up, identifying as Black was associated with a lower risk of a severe liver outcome (HR 0.55; 95%CI 0.44–0.66) in the fully adjusted model, and there was no significant association between racial identifier and severe liver disease in patients without a CLD diagnosis during follow-up (HR 1.15; 95%CI 0.77–1.71; S1 Fig and S3 Table).

**Table 3:** Cox regression models, stratified by time, examining the association of race as the primary predictor variable for the outcome of time to a severe liver disease diagnosis.

	Model 1	Model 2	Model 3	Model 4	Model 5
	HR	HR	HR	HR	HR
**Covariates**	95% CI	95% CI	95% CI	95% CI	95% CI
Black	1.32 1.13–1.54	1.11 0.95–1.30	0.93 0.80–1.09	0.90 0.76–1.06	0.68 0.57-0.81
CLD		22.97 18.99–27.79	17.13 14.10–20.82	16.58 13.57–20.25	13.80 11.12-17.13
FIB-4 Scores at Baseline					
1.3 ≤ FIB-4 < 2.67			3.02 2.43–3.75	3.00 2.41–3.74	2.73 2.19–3.41
FIB-4 ≥ 2.67			10.88 8.84–13.39	9.48 7.67–11.72	7.61 6.13–9.45
Male				0.92 0.79–1.08	0.83 0.71–0.98
Unmarried				1.35 1.13–1.61	1.32 1.10–1.57
Smoker				0.97 0.80–1.17	0.94 0.78–1.14
Poverty status				0.94 0.79–1.11	0.96 0.81–1.14
Remote residence				1.15 0.93–1.41	1.17 0.95–1.45
BMI					0.99 0.98–1.01
Hypertension					1.21 0.96–1.54
Diabetes					1.59 1.33–1.89
Hyperlipidemia					0.73 0.61–0.87
CVD					0.96 0.81–1.13
CKD					2.17 1.82–2.59
**Alcohol use disorder**					1.41 1.16–1.70

HR = hazard ratio. CI = confidence interval. BMI = body mass index. CKD = chronic kidney disease. CLD = chronic liver disease. CVD = cardiovascular disease. FIB-4 = Fibrosis-4 Index. Model 1 is the unadjusted model; Model 2 adjusts for any known diagnosis of CLD; Model 3 adjusts for any diagnosed CLD and baseline advanced fibrosis risk by FIB-4 score; Model 4 is Model 3 plus adjustment for other potentially confounding covariates including demographic and comorbidity variables.

## Discussion

Using a robust cohort in primary care that included a large proportion of patients identified as Black, we demonstrated that after adjusting for known chronic liver disease, baseline fibrosis risk, demographic, and comorbidity variables, identification as Black was associated with a significantly lower hazard of a severe liver disease outcome compared to patients identified as non-Black (HR 0.66; 95%CI 0.55–0.79). The association between identification as Black and the occurrence of a severe liver disease outcome evolved from a positive association with severe liver disease (HR 1.32) to “protective” (HR 0.66) with each series of covariate adjustments. In the subset of patients with known diagnoses of CLD during follow-up, identifying as Black was also associated with a lower hazard of a severe liver disease diagnosis (HR 0.53; 95%CI 0.44–0.65). The E-value of 2.30 (95%CI 1.76–2.90) suggests that substantial unmeasured confounding would be necessary to explain away the estimate of the association between identification as Black and time to a severe liver disease diagnosis. This relationship reinforces the health disparities within our healthcare system and heightens the urgency to improve care delivery. These findings emphasize the complexity of race as a construct constituting not only biologic factors, but incorporating factors of bias, discrimination, restrictions in healthcare access, and other historical care disparities [13]. When other demographic and comorbidity variables are controlled for in this analysis, patients identified as Black have a lower hazard of receiving a diagnosis for a severe liver disease outcome. In the raw data, without covariate adjustment, this potential “advantage” disappears. Hypertension, diabetes mellitus, and chronic kidney disease are all associated with severe liver disease outcomes, and all three of these chronic conditions disproportionately impact Black patients and are well-known drivers of healthcare disparities [28–30]. Thus, we focused on reducing the potentially distortive influence of demographic and comorbidity variables have on the relationship between race and severe liver disease outcomes [31]. Targeted efforts to improve the management of the chronic conditions, including chronic liver disease, will be necessary to achieve health equity.

These findings are consistent with previous data that suggest that there may be a lower risk for progressing to cirrhosis in Black patients with known CLD, specifically HCV and NAFLD [9,32–34]. In a study of over 5,000 U.S. Veterans undergoing liver stiffness measurement (LSM), a lower proportion of patients with LSMs at risk for advanced fibrosis were identified as non-Hispanic Black compared to Veterans identified as non-Hispanic White and Hispanic [35]. Another study of 31,000 patients with NAFLD demonstrated a significantly lower risk of incident cirrhosis in patients identified as Black (HR 0.34) [36]. Such data raise the possibility that there may be a genetic explanation or contribution to the relationship between Black race and progression to cirrhosis [37,38]. Work investigating the relationship between metabolic dysfunction, genetics, and fibrosis risk in ALD demonstrated a link between genetic makeup and a heightened risk of liver fibrosis stage and liver inflammation [39]. A NAFLD study explored the relationship of genetic risk factors, alleles for PNPLA3 and HSD17B13, which demonstrated that these genetic risks augmented the relationship between metabolic risk factors and advanced fibrosis [40]. Future work dedicated to the exploration of pathways of CLD progression to fibrosis and cirrhosis is warranted.

A remarkable finding of this study was the intersection and crossing of the severe liver disease outcome curves by race over time (**Fig 2**). This violated the assumption of proportional hazards and forced us to modify our statistical analysis plan [41–43]. The cause(s) of the intersection of these outcome curves is (are) not known but could be related to the influence of other factors (e.g., comorbidities, social determinants of health, access to care) on severe liver disease outcomes over time. It is also necessary to consider that the significantly lower hazard of experiencing a severe liver disease event in Black patients is really a feature of delayed identification and diagnosis of cirrhosis and its complications. Because this study relies upon the identification of a composite of ICD-9/10 diagnosis codes for severe liver event ascertainment, it is limited by any factors that would contribute to diagnostic delay, including reduced access to care, bias, or discrimination [44]. If this is the case, concerted efforts are needed to strengthen primary care, improve primary care engagement for all patients, and increase health system accountability for improving the timeliness of severe liver disease diagnoses in all patients.

Enhancing primary care delivery for all patients with CLD, and risk factors for CLD, to address disparities in care and diagnostic errors will require inter-disciplinary strategies and multi-dimensional approaches [45]. For viral hepatitis, systematic HCV and HBV screening can improve CLD detection, but increased diagnosis needs to be coupled with improved and equitable access to anti-viral therapies to avoid exacerbating care gaps [46,47]. For example, it has been shown that expansion of HCV anti-viral prescribing from specialty care to primary care and from hospital-based clinics to community settings can improve cure rates for all affected patients [48]. In this study, primary care clinicians, in combination with clinic pharmacists, prescribed anti-viral therapies for all patients with HCV (patients with HCV cirrhosis receive therapy from Hepatology), and patients with HBV are referred to Infectious Disease and Hepatology specialists for prescription of therapy. Primary care providers also play a central role in the diagnosis and management of alcohol-related liver disease. Expanded and consistent implementation of alcohol use screening in primary care with validated surveys (e.g., AUDIT-C) and laboratory tests (e.g., phosphatidylethanol [PEth]) can significantly enhance the identification of at-risk alcohol use and connection to alcohol use support services and medication-assisted treatment (e.g., naltrexone) [49–51]. Finally, recently issued guidelines on the management of MASLD emphasize the importance of primary care clinicians for identifying MASLD diagnoses, assessing advanced fibrosis risk, and treating MASLD through weight loss and cardiovascular risk reduction interventions [52,53]. Expansion of CLD diagnosis, management, and surveillance interventions into the primary care space holds the potential to significantly improve CLD care for all patients and reduce care delays that result in severe liver disease events.

We recognize limitations of this study. First, examination of race is an extremely complex construct and its study is limited by features of structural racism that contribute to healthcare disparities [54]. Further, racial identifiers incorporate environmental factors, socioeconomic status, discrimination, and often, restricted access to care [13]. These factors cannot be fully captured by our data. Also, this study does rely upon ICD-9/10 diagnosis codes, which may contribute to possible misclassification bias. Another limitation is that this is a single center, which threatens generalizability; however, a clear strength of the study is that this study included a large proportion of Black patients, making it more generalizable to the southeastern U.S. population than previously published studies with very small proportions of Black patients [55].

## Conclusion

After adjusting for confounding covariates, patients identified as Black had a longer time to receiving a diagnosis of severe liver disease compared to non-Black patients. This raises the possibility that there is significant delay in the detection of severe liver disease in patients identified as Black, or that there may be an underlying biological fibrosis program that differs between patients identified as Black compared to their non-Black counterparts. Opportunity exists to better understand fibrosis pathways in this population and every effort must be made to narrow current healthcare disparities.

## Supporting information

S1 Table ICD-9/10 codes for severe liver disease outcomes and chronic liver disease covariates. (DOCX)

S2 Table Cox regression models, stratified by time, examining race as the primary predictor variable for the outcome of SLD. Adjustment by one additional covariate occurs in each successive model.
(DOCX)

S3 Table Cox regression models examining race as the primary predictor variable for the outcome of severe liver disease in patients with known chronic liver disease diagnoses and those patients with no known chronic liver disease diagnosis during follow-up. (DOCX)

S1 Fig Kaplan Meier survival curves for the outcome of time to a severe liver disease diagnosis (cirrhosis, complication of cirrhosis, or hepatocellular carcinoma) by race for patients with a known CLD diagnosis (A) and those patients without a diagnosis of CLD during follow-up (B). (TIF)
