# Supplementary material for: The association of race with time to severe liver disease diagnoses
Source: PLoS One. 2025 Oct 14;20(10):e0334016. doi: 10.1371/journal.pone.0334016 (PMC12520358; doi:10.1371/journal.pone.0334016)
Supplement: S1 Table — (DOCX) [file pone.0334016.s001.docx]

**Supplement**

**Supplementary Table S1.** ICD-9/10 codes for severe liver disease outcomes and chronic liver disease covariates.

| Severe liver disease outcomes | ICD-10 | ICD-9 |
| --- | --- | --- |
| Cirrhosis | K65.2, K70.11, K70.30, K70.31, K72.10, K72.11, K74.4-K74.5, K74.60, K74.69, K76.7, I85.00, I85.01, I85.10, I85.11, I86.4, R18.8 | 456.0, 456.1, 456.2, 456.21, 567.23, 571.2, 571.5, 572.2-572.4, 789.5 |
| Ascites | R18.8 | 789.5 |
| Hepatic encephalopathy | K72.11 | 572.2 |
| Hepatorenal syndrome | K76.7 | 572.4 |
| Portal hypertension | K76.6 | 572.3 |
| Varices | I85.00, I85.01, I85.10, I85.11 | 456.0, 456.1, 456.2, 456.21 |
| Hepatocellular carcinoma | C22.0 | 155.0 |
| History of liver transplantation | Z94.4 | V42.7 |
| Chronic liver disease diagnoses |  |  |
| Viral hepatitis B | B16.0, B16.1, B18.0, B18.1 | 070.20 - 070.23, 070.30 - 070.33 |
| Viral hepatitis C | B17.10, B17.11, B18.2, B19.20, B19.21 | 070.41, 070.44, 070.51, 070.54, 070.70, 070.71 |
| Alcohol-related liver disease (and alcohol use disorder) | F10, E52, G62.1, I42.6, K29.2, K70.0, K70.3, K70.9, T51.x, Z50.2, Z71.4, Z72.1 | 265.2, 291.1-291.3, 291.5-291.9, 303.0, 303.9, 305.0, 357.5, 425.5, 535.3, 571.0-571.3, 980.x, V11.3 |
| Nonalcoholic fatty liver disease | K75.81, K76.0 | 571.8 |
| Autoimmune hepatitis | K75.4 | 571.42 |
| Primary biliary cholangitis | K74.3 | 571.6 |
| Hemochromatosis | E83.110, E83.111, E83.118, E83.119 | 275.01-275.03, 238.72 |
| Wilson’s disease | E83.01 | 275.1 |
| α-1 anti-trypsin deficiency | E88.01 | 273.4 |

ICD=International Classification of Diseases.
