# Supplementary material for: The association of race with time to severe liver disease diagnoses
Source: PLoS One. 2025 Oct 14;20(10):e0334016. doi: 10.1371/journal.pone.0334016 (PMC12520358; doi:10.1371/journal.pone.0334016)
Supplement: S2 Table — Adjustment by one additional covariate occurs in each successive model. (DOCX) [file pone.0334016.s002.docx]

**Supplementary Table S2.** Cox regression models, stratified by time, examining race as the primary predictor variable for the outcome of SLD. Adjustment by one additional covariate occurs in each successive model.

|  | Model 1 | Model 2 | Model 3 | Model 4 | Model 5 |
| --- | --- | --- | --- | --- | --- |
| Covariates | HR  (95%CI) | HR  (95%CI) | HR  (95%CI) | HR  (95%CI) | HR  (95%CI) |
| Black | 1.32  (1.13-1.53) | 1.11  (0.95-1.29) | 0.93  (0.80-1.08) | 0.95  (0.81-1.11) | 0.88  (0.75-1.03) |
| CLD |  | 22.97  (18.99-27.79) | 17.13  (14.1-20.82) | 16.9  (13.88-20.58) | 16.46  (13.5-20.06) |
| FIB-4 Indet. |  |  | 3.02  (2.43-3.75) | 2.95  (2.37-3.67) | 2.98  (2.4-3.71) |
| FIB-4 High |  |  | 10.88  (8.84-13.39) | 9.49  (7.68-11.72) | 9.45  (7.65-11.67) |
| Male |  |  |  | 0.89  (0.76-1.03) | 0.91  (0.78-1.06) |
| Unmarried |  |  |  |  | 1.33  (1.12-1.58) |
|  | **Model 6** | **Model 7** | **Model 8** | **Model 9** | **Model 10** |
| Black | 0.88  (0.75-1.03) | 0.89  (0.75-1.04) | 0.90  (0.76-1.06) | 0.90  (0.76-1.06) | 0.84  (0.71-1.00) |
| CLD | 16.57  (13.57-20.24) | 16.56  (13.56-20.22) | 16.59  (13.58-20.26) | 16.58  (13.57-20.25) | 16.15  (13.22-19.73) |
| FIB-4 Indet. | 2.98  (2.40-3.71) | 2.99  (2.41-3.72) | 3.00  (2.41-3.73) | 3.00  (2.41-3.74) | 2.80  (2.24-3.49) |
| FIB-4 High | 9.46  (7.66-11.68) | 9.44  (7.65-11.66) | 9.47  (7.67-11.69) | 9.48  (7.67-11.72) | 8.75  (7.06-10.85) |
| Male | 0.91  (0.78-1.07) | 0.92  (0.78-1.08) | 0.92  (0.79-1.08) | 0.92  (0.79-1.08) | 0.91  (0.77-1.06) |
| Unmarried | 1.34  (1.12-1.59) | 1.34  (1.12-1.59) | 1.35  (1.13-1.61) | 1.35  (1.13-1.61) | 1.36  (1.14-1.63) |
| Smoking | 0.96  (0.80-1.15) | 0.96  (0.80-1.15) | 0.97  (0.80-1.16) | 0.97  (0.80-1.17) | 0.96  (0.80-1.16) |
| Remote residence |  | 1.14  (0.93-1.40) | 1.15  (0.93-1.41) | 1.15  (0.93-1.41) | 1.15  (0.94-1.42) |
| Poverty |  |  | 0.94  (0.79-1.11) | 0.94  (0.79-1.11) | 0.93  (0.78-1.10) |
| BMI |  |  |  | 1.00  (0.99-1.01) | 1.00  (0.99-1.01) |
| Hypertension |  |  |  |  | 1.51  (1.22-1.88) |
|  | **Model 11** | **Model 12** | **Model 13** | **Model 14** | **Model 15** |
| Black | 0.77  (0.65-0.92) | 0.76  (0.64-0.9) | 0.76  (0.64-0.9) | 0.66  (0.55-0.79) | 0.68  0.57-0.81 |
| CLD | 15.66  (12.82-19.13) | 15.49  (12.68-18.93) | 15.45  (12.65-18.88) | 15.80  (12.93-19.3) | 13.80  11.12-17.13 |
| FIB-4 Indet. | 2.74  (2.2-3.42) | 2.84  (2.27-3.54) | 2.82  (2.26-3.53) | 2.74  (2.19-3.42) | 2.73  2.19-3.41 |
| FIB-4 High | 8.39  (6.76-10.4) | 8.49  (6.85-10.54) | 8.48  (6.84-10.52) | 7.93  (6.4-9.84) | 7.61  6.13-9.45 |
| Male | 0.91  (0.78-1.07) | 0.92  (0.78-1.08) | 0.92  (0.78-1.08) | 0.88  (0.75-1.03) | 0.83  0.71-0.98 |
| Unmarried | 1.41  (1.18-1.68) | 1.38  (1.16-1.65) | 1.39  (1.16-1.65) | 1.35  (1.13-1.61) | 1.32  1.10-1.57 |
| Smoking | 0.97  (0.8-1.16) | 0.93  (0.77-1.12) | 0.93  (0.77-1.12) | 1.01  (0.84-1.22) | 0.94  0.78-1.14 |
| Remote residence | 1.17  (0.95-1.44) | 1.13  (0.92-1.4) | 1.14  (0.92-1.4) | 1.13  (0.92-1.39) | 0.96  0.81-1.14 |
| Poverty | 0.95  (0.8-1.13) | 0.95  (0.8-1.13) | 0.95  (0.8-1.13) | 0.98  (0.83-1.16) | 1.17  0.95-1.45 |
| BMI | 0.99  (0.98-1) | 0.99  (0.98-1) | 0.99  (0.98-1) | 0.99  (0.98-1) | 0.99  0.98-1.01 |
| Hypertension | 1.28  (1.03-1.61) | 1.39  (1.1-1.75) | 1.38  (1.09-1.74) | 1.24  (0.98-1.57) | 1.21  0.96-1.54 |
| Diabetes | 1.73  (1.47-2.05) | 1.81  (1.53-2.15) | 1.8  (1.51-2.13) | 1.56  (1.31-1.86) | 1.59  1.33-1.89 |
| Hyperlipidemia |  | 0.79  (0.66-0.94) | 0.78  (0.66-0.93) | 0.72  (0.6-0.86) | 0.73  0.61-0.87 |
| CVD |  |  | 1.07  (0.91-1.26) | 0.99  (0.84-1.17) | 0.96  0.81-1.13 |
| CKD |  |  |  | 2.18  (1.83-2.6) | 2.17  1.82-2.59 |
| Alcohol use disorder |  |  |  |  | 1.41  1.16-1.70 |

HR=hazard ratio. CI=confidence interval. CLD=chronic liver disease. BMI=body mass index. CVD=cardiovascular disease. CKD=chronic kidney disease.
