# Supplementary material for: The association of race with time to severe liver disease diagnoses
Source: PLoS One. 2025 Oct 14;20(10):e0334016. doi: 10.1371/journal.pone.0334016 (PMC12520358; doi:10.1371/journal.pone.0334016)
Supplement: S3 Table — (DOCX) [file pone.0334016.s003.docx]

**Supplementary Table S3**. Cox regression models examining race as the primary predictor variable for the outcome of severe liver disease in patients with known chronic liver disease diagnoses and those patients with no known chronic liver disease diagnosis during follow-up.

|  | Known CLD Diagnosis | | No Known CLD Diagnosis During Follow-up | |
| --- | --- | --- | --- | --- |
|  | Unadjusted | Fully adjusted | Unadjusted | Fully adjusted |
| Variables | HR (95%CI) | HR (95%CI) | HR (95%CI) | HR (95%CI) |
| Black | 0.79  (0.67-0.94) | 0.55  (0.45-0.66) | 1.89  (1.34-2.68) | 1.15  (0.77-1.71) |
| FIB-4 Indet. |  | 3.33  (2.56-4.34) |  | 1.86  (1.23-2.82) |
| FIB-4 High |  | 12.02  (9.38-15.42) |  | 4.84  (2.99-7.82) |
| Male |  | 0.80  (0.67-0.95) |  | 1.02  (0.71-1.48) |
| Unmarried |  | 1.29  (1.05-1.57) |  | 1.09  (0.74-1.61) |
| Smoking |  | 1.00  (0.82-1.23) |  | 1.39  (0.83-2.33) |
| Remote residence |  | 1.24  (0.98-1.57) |  | 1.41  (0.88-2.27) |
| Poverty |  | 0.94  (0.78-1.14) |  | 0.98  (0.68-1.42) |
| BMI |  | 0.99  (0.98-1.01) |  | 0.99  (0.96-1.01) |
| Hypertension |  | 1.06  (0.82-1.36) |  | 1.62  (0.89-2.95) |
| Diabetes |  | 1.81  (1.49-2.20) |  | 1.52  (1.02-2.26) |
| Hyperlipidemia |  | 0.60  (0.49-0.73) |  | 0.62  (0.41-0.93) |
| CVD |  | 0.98  (0.82-1.18) |  | 0.90  (0.62-1.31) |
| CKD |  | 1.86  (1.53-2.26) |  | 3.78  (2.53-5.64) |
| Alcohol use disorder |  | 1.34  (1.11-1.64) |  | 3.69  (2.26-6.03) |

HR=hazard ratio. CI=confidence interval. CLD=chronic liver disease. BMI=body mass index. CVD=cardiovascular disease. CKD=chronic kidney disease.
