# Supplementary figures and images for: The association of race with time to severe liver disease diagnoses
Source: PLoS One. 2025 Oct 14;20(10):e0334016. doi: 10.1371/journal.pone.0334016 (PMC12520358; doi:10.1371/journal.pone.0334016)

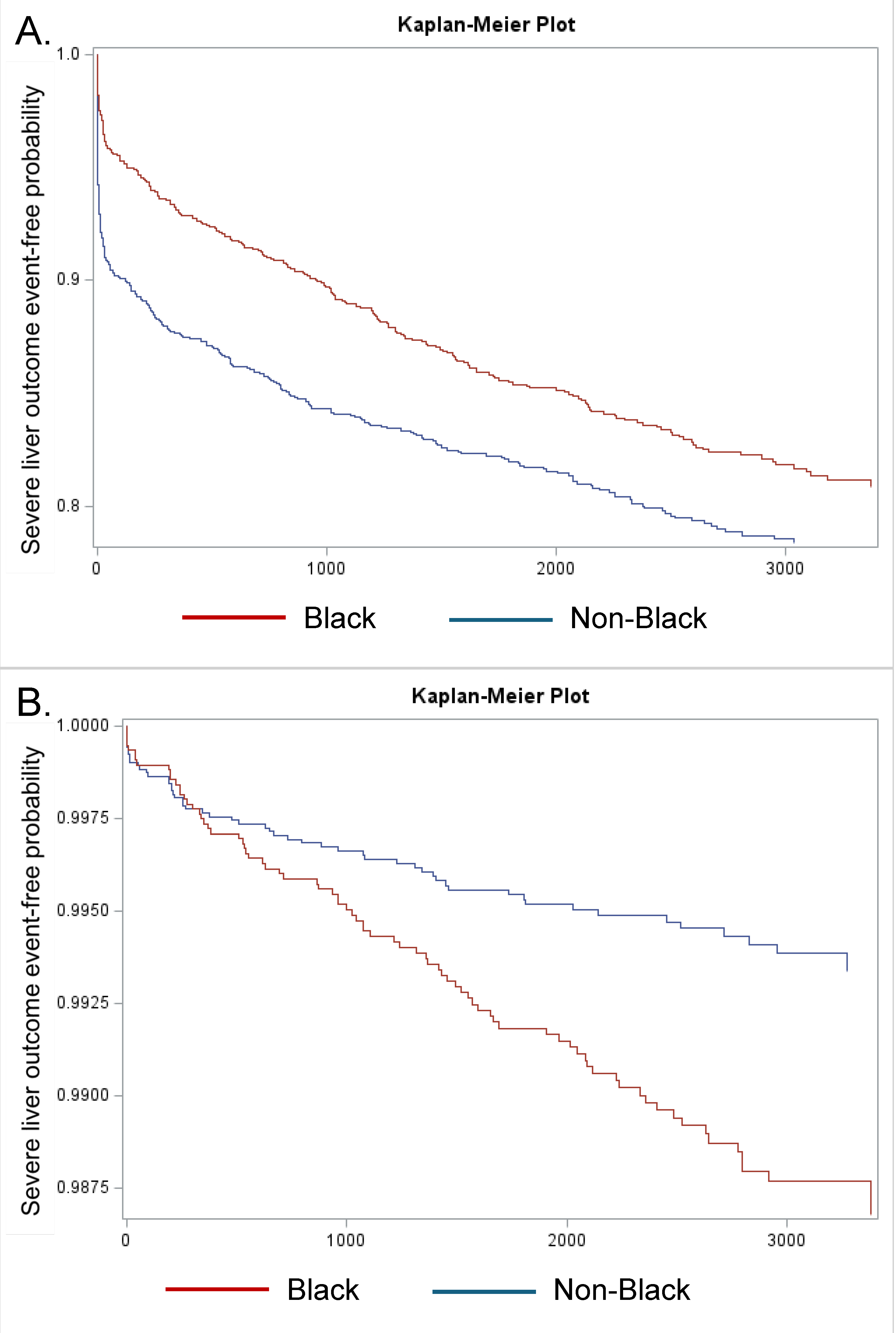

Supplement: S1 Fig — (TIF) [file pone.0334016.s004.tif]
